# Prevalence of common carbapenemase genes and multidrug resistance among uropathogenic Escherichia coli phylogroup B2 isolates from outpatients in Wasit Province/ Iraq

**DOI:** 10.1371/journal.pone.0262984

**Published:** 2022-01-25

**Authors:** Sareaa Maseer Gatya Al-Mayahie, Dhifar Raa’d Taher Al-Guranie, Aya Aziz Hussein, Zaineb Ali Bachai

**Affiliations:** Department of Biology, College of Science, Wasit University, Al-Kut City, Wasit Province, Iraq; Suez Canal University, EGYPT

## Abstract

Carbapenems are the last resort antimicrobials for the treatment of extended spectrum β-lactamases (ESBLs) producing Enterobacteriaceae. Emergence of carbapenems resistant group B2 uropathogenic *E*. *coli* (UPEC) is a major concern because of their high virulence. Prevalence of these enzymes and multidrug resistance (MDR) among B2 UPEC isolates from Iraqi outpatients with acute urinary tract infection (UTI) was evaluated in this research. Urine cultures were performed and the isolates were identified biochemically. *Escherichia coli* isolates were tested for phylogroup reference by quadraplex PCR, then B2 isolates were detected for antimicrobial resistance by disc diffusion test and carbapenemase genes by PCR. *Escherichia coli* was the most prevalent among Gram-negative isolates (66.6%) and B2 was the most detected phylogroup among *E*. *coli* isolates (33.9%). Most of B2 isolates showed high resistance rates to tested antimicrobials, especially β-lactams with MDR revealed in 100% of them. Whereas, low resistance rates were noted against carbapenems, aminoglycosides and nitrofurantoin. Carbapenemase genes were detected in 76.3% of B2 isolates. Of which, *bla*_OXA-48_ was the most frequent (57.8%), followed by *bla*_PER_ (47.3%), *bla*_KPC_ (15.7%), *bla*_VEB_ and *bla*_VIM_ (10.5%, for each). Whereas, *bla*_GES_ and *bla*_IMP_ genes were not found. Coproduction of these genes occurred among 17 isolates. The combination of *bla*_OXA-48_ and *bla*_PER_ was the most frequent (41.1%). All carbapenemase producing isolates were MDR. These results revealed high prevalence of carbapenemase genes and MDR among B2 UPEC recovered in this study. In the study area. it is strongly advised to use aminoglycosides and nitrofurantoin for empirical treatment of UPEC.

## Introduction

*Escherichia coli* (*E*. *coli*) is the most versatile known microorganism [1]. It is a common commensal of gastrointestinal tract of human and animal. Also, it comprises pathogenic strains divided into intestinal or diarrhoeagenic *E*. *coli* (DEC) and extraintestinal pathogenic *E*. *coli* (ExPEC). The main infections caused by ExPEC are urinary tract infections (UTIs), sepsis, meningitis, and wound infections [2–4]. A large proportion of humans are affected by UTIs, with annual prevalence of about 150 million cases. Yearly, $5 billion are spent for treatment of UTIs in the USA with estimated cases of 11 million per year [5]. Uropathogenic *E*. *coli* is the most prevalent ExPEC and it is the primary cause of UTIs all over the world [3]. Uropathogenic *E*. *coli* strains have a wide variety of virulence factors which include: immune suppressors, adhesins (fimbrial and afimbrial adhesins), siderophore systems, the serum resistance, the capsular polysaccharide K antigen, and toxins [3, 5].

Carbapenems emerged as bactericidal β-lactam antimicrobials with confirmed activity in severe infections caused by extended spectrum β-lactamases (ESBLs) producers [6]. These antimicrobials have broad spectrum antibacterial activity and have a unique structure that is composed of a carbapenem linked to a β-lactam ring which provides protection against most β lactamases such as metallo-β-lactamases (MBLs) and ESBLs [7]. Therefore; carbapenems are considered one of the most reliable drugs for treating bacterial infections and the occurrence and distribution of resistance to them constitute a major public health problem [8]. There are three possible mechanisms for carbapenems’ resistance in the family Enterobacteriaceae. These are: efflux pump overactivity, porin loss or mutation, and carbapenemase production, which is the main mechanism of resistance to these antimicrobials [9–11]. Carbapenemases are enzymes (β-lactamases) that are encoded by both chromosomal and plasmid-mediated genes and structurally belonging to different Ambler classes (A, B, and D). These enzymes can hydrolyze a broad range of β-lactams, including carbapenems, cephalosporins, penicillin, and aztreonam. Also, bacterial strains possessing carbapenemases are often resistant to multiple drugs (MDR) [11]. Most of these enzymes have been mainly found in Enterobacteriaceae, *Pseudomonas aeruginosa*, and *Acinetobacter baumannii*. For the Enterobacteriaceae family, class A carbapenemases (KPC enzymes) appeared in North Carolina (USA) in 1996 and then spread to Europe; class B discovered as VIM-1 in *E*. *coli* in Greece, but rapidly spread in *Klebsiella pneumoniae* (*K*. *pneumoniae*), becoming endemic in that country as well as in other European countries; and OXA-48 (class D) which was first reported in Turkey in *K*. *pneumoniae*, and later occurred in other Mediterranean countries [8, 11]. Nowadays, carbapenemases in Enterobacteriaceae are mainly found in *K*. *pneumoniae*, and to a much lesser extent in *Escherichia coli* (*E*. *coli*) and other enterobacterial species, with a higher prevalence in southern Europe and Asia than in other parts of the world. It has recently been concluded that global spread of carbapenems-resistant enterobacterial isolates (CRE) in the future will be dominated in the hospital environment by *K*. *pneumoniae* producing all types of carbapenemases, mainly KPC, VIM, NDM, and OXA-48, and in the community by *E*. *coli* having NDM or OXA-type (OXA-48 and OXA- 181) enzymes [11].

Multidrug resistance has been increased all over the world that is considered a public health threat. Several recent investigations reported the emergence of multidrug-resistant bacterial pathogens from different origins including humans, birds, cattle, and fish that increase the need for routine application of the antimicrobial susceptibility testing to detect the antibiotic of choice as well as the screening of the emerging MDR strains [12–15]. Worldwide, there is a major concern regarding the high prevalence of antimicrobial resistance and MDR among UPEC. By 2050, it has been estimated that more than 3 million people may lose their lives each year as a result of increase in MDR. A major concern in this respect is the spread of carbapenem-resistant strains all over the world [3].

Strains of *E*. *coli* are divided into eight phylogroups (A, B1, B2, C, D, E, F and clade I) of which group B2 strains are the most virulent and the most common causative agents of UTI [16, 17]. Emergence of carbapenem resistant group B2 UPEC is a major concern because of their high virulence. Strains with high antimicrobial resistance accompanied by high virulence may emerge and this may lead to treatment failure and loss of effective treatment [10]. So that, awareness should be taken to prevent such strains from reaching our patients especially those who are immunocompromised. One of the effective measurements to achieve this aim is to do periodic surveillance of antimicrobial resistance of UPEC by both phenotypic and genotypic procedures. Selection of appropriate prevention and containment options requires good knowledge of the prevalence and incidence of carbapenemases. Strains-producing these enzymes are not limited to hospital environment, but also occur among hospitals, long-term care facilities, community, animals, and the environment [11]. As it is difficult to develop novel antimicrobial agents, efforts should be concentrated on the prevention of the spread of carbapenemase producers by early detection and reinforced hygiene measures [18] and careful monitoring of use of antimicrobials for UTI treatment is necessary [19]. In addition, predominance of carbapenemases among B2 *E*. *coli* isolates was reported from different parts of the world [20–23]. Hence, this research was designed to evaluate the prevalence of carbapenemase genes and MDR among B2 UPEC isolates from Iraqi outpatients in Wasit Province with acute UTI by multiplex PCR protocols.

## Materials and methods

### Specimen collection and processing

Midstream urine samples were collected from outpatients attending AlKarama hospital and Al-Kut hospital for Gynecology and Obstetrics and Pediatrics in Al-Kut/Wasit Province/Iraq, during the period from July, 2018 to January, 2019. The urine samples were collected into sterile screw capped test tubes and streaked immediately on MacConkey agar (HIMEDIA, India) and Blood agar (HIMEDIA, India) plates for bacterial isolation [24].

### Isolation, identification and phylogenetic grouping of *E*. *coli*

Collected urine samples were cultured immediately after collection on MacConkey agar and Blood agar plates and incubated aerobically at 37°C for 24 h. Positive urine cultures were defined by a growth of single morphotype of colony with counts >10^5^CFU/ml [24]. From each plate single colony (with the appropriate color and morphology, that is, characteristics of *E*. *coli*) was selected and subcultured onto MacConkey agar plate again, incubated, a single colony was selected and subcultured onto tryptic soy agar (TSA) (HIMEDIA, India) plate, and then kept in refrigerator for further work.

The isolates were identified biochemically by API20E rapid test system depending on the manufacturer’s instructions (BioMerieux, France).

Thereafter, *E*. *coli* isolates were classified into eight phylogenetic groups (A, B1, B2, C, D, E, F, and clade I) according to the presence of *chuA*, *yjaA*, and *arpA* genes and TspE4.C2 DNA fragment, depending on methods provided by Clermont *et al*. [17] At first the DNA was extracted from the isolates by boiling method described by Yamamoto *et al*. [25] with modification which included suspending 24 hr. old bacterial culture (3 loopfuls) on TSA in 1 ml of sterile 1X TE buffer (pH 8.0) (Bio Basic, Canada) instead of sterile D.W. The cell suspension was boiled in water bath at 95˚C for 10 minutes. The suspension was centrifuged at 10,000 rpm for 5 minutes. The supernatant which contains purified DNA was dispensed in 100 μl aliquots and stored at -20˚C till use. The phylogroups were determined by quadraplex PCR (Tables 1 and 2).

**Table 1:** Primer’s sequence for detection of *E*. *coli* phylogroups.

Gene	Primer name	Primer sequence (5′- 3′)	Product size (bp)	Source of primer
*chuA*	chuA. 1b	ATGGTACCGGACGAACCAAC	288	Clermont *et al*. [17]; Clermont *et al*. [26]
	chuA. 2b	TGCCGCCAGTACCAAAGACA		
*yjaA*	yjaA. 1b	CAAACGTGAAGTGTCAGGAG	211	Clermont *et al*. [17]
	yjaA. 2b	AATGCGTTCCTCAACCTGTG		
TspE4.C2	TspE4.C2.1b	CACTATTCGTAAGGTCATCC	152	Clermont *et al*. [17]
	TspE4C2. 2b	AGTTTATCGCTGCGGGTCGC		
*arpA*	AceK.f	AACGCTATTCGCCAGCTTGC	400	Clermont *et al*. [17]; Clermont *et al*. [27]
	ArpA1.r	TCTCCCCATACCGTACGCTA		
*arpA*	ArpAgpE.f	GATTCCATCTTGTCAAAATATGCC	301	Lescat *et al*. [28]
	ArpAgpE.r	GAAAAGAAAAAGAATTCCCAAGAG		
*trpA*	trpAgpC.1	AGTTTTATGCCCAGTGCGAG	219	Lescat *et al*. [28]
	trpAgpC.2	TCTGCGCCGGTCACGCCC		
*trpA*	trpBA.f	CGGCGATAAAGACATCTTCAC	489	Clermont *et al*. [29]
	trpBA.r	GCAACGCGGCCTGGCGGAAG		

**Table 2:** Components of 50μl PCR master mix and amplification conditions for detection of UPEC phylogroups [17].

PCR reaction	SterileD.W.	Primers	DNA	Amplification conditions
**Quadruplex**	38 μl	8 μl: 1 μl each of:	5 μl	1. Initial denaturation at 94°C for 4 min. 2. 30 cycles of: • Denaturation at 94°C for 5 s. • Annealing at 57°C (group E) or 59°C (quadruplex and group C) for 20 s. • Extension at 72°C for 1 min.
		• chuA-1b, chuA-2b		
		• yiaA.1b, yiaA.2b		
		• TspE4C2.1b,		
		TspE4C2.2b • Acek.f, ArpA.r		
**Group E**	41 μl	4 μl: 1 μl each of:	5 μl	3. Final extension at 72°C for 5 min.
		• ArpAgpE.f, ArpAgpE.r		
		• trpBA.f, trpBA.r		
**Group C**	41 μl	4 μl: 1 μl each of:	5 μl	
		• trpAgpC.1, trpAgpC.2		
		• trpBA.f, trpBA.r		

This work was approved by the Scientific Committee of the College of Science/ Wasit University/ Wasit Province/ Iraq (Scientific project discussing committee) and also it was performed after obtaining permission from Health Administration of Wasit, Wasit Province/Iraq. Oral consent was obtained from each patient for collecting specimens and publication of this report. The reason for just obtaining oral consent without the need for written consent is that collection of urine specimens is part of routine clinical laboratory work for diagnosis of these infections. All patients’ data were anonymous.

### Antimicrobial susceptibility of the isolates

Antimicrobial susceptibility of the isolates was performed by the disk diffusion method according to the instructions of CLSI [30] using Mueller-Hinton agar (HIMEDIA, India). The used antimicrobials were obtained from Bioanalyse/ Turkey and included: ampicillin (AMP: 10 *μ*g); amoxicillin-clavulanic acid (AMC: 20/10 *μ*g); cefoxitin (FOX: 30 *μ*g); cefotaxime (CTX: 30 *μ*g); ceftazidime (CAZ: 30 *μ*g); ceftriaxone (CRO: 30 *μ*g); cefepime (FEP: 30 *μ*g); aztreonam (ATM: 30 *μ*g); imipenem (IPM: 10 *μ*g); meropenem (MEM: 10 *μ*g); gentamicin (CN: 10 *μ*g); amikacin (AK: 30 *μ*g); tetracycline (TE: 30 *μ*g); nalidixic acid (NA: 30 *μ*g); ciprofloxacin (CIP: 5 *μ*g); trimethoprim-sulfamethoxazole (SXT: 1.25/23.75 *μ*g); and nitrofurantoin (F: 300 *μ*g).

### Molecular detection of carbapenemase genes

Protocols for multiplex PCR that were developed by Dallenne *et al*. [31] were followed for amplification of carbapenemase genes. For each primer (Table 3), 100 μl of working solution was prepared by diluting the stock solution (100 pmol/μl) by 1X TE buffer ((Bio Basic, Canada)) (pH 8.0) depending on the general dilution equation: C1V1 = C2V2. So that 1μl of forward primer and 1μl of reverse primer of each gene should contain the appropriate concentration to be added to the PCR master mix (final volume 50 μl). Primer’s concentrations ranged from 10 pmole/μl to 25 pmole/μl (Table 4).

**Table 3:** Primers’ sequence of carbapenemase genes [31].

Multiplex PCR pool	Primer name	Sequence (5’-3’)	Amplicon size (bp)
**Multiplex-I**: *bla*_VEB_, *bla*_PER_ and *bla*_GES_	MultiGES-F	AGTCGGCTAGACCGGAAAG	399
	MultiGES-R	TTTGTCCGTGCTCAGGAT	
	MultiPER-F	GCTCCGATAATGAAAGCGT	520
	MultiPER-R	TTCGGCTTGACTCGGCTGA	
	MultiVEB-F	CATTTCCCGATGCAAAGCGT	648
	MultiVEB-R	CGAAGTTTCTTTGGACTCTG	
**Multiplex-II**: *bla*_GES_ and *bla*_OXA-48-like_	MultiGES-F	AGTCGGCTAGACCGGAAAG	399
	MultiGES-R	TTTGTCCGTGCTCAGGAT	
	MultiOXA-48-F	GCTTGATCGCCCTCGATT	281
	MultiOXA-48-R	GATTTGCTCCGTGGCCGAAA	
**Multiplex III:** *bla*_IMP_, *bla*_VIM_, and *bla*_KPC_	MultiIMP-F	TTGACACTCCATTTACDGb	139
	MultiIMP-R	GATYGAGAATTAAGCCACYCTb	
	MultiVIM-F	GATGGTGTTTGGTCGCATA	390
	MultiVIM- R	CGAATGCGCAGCACCAG	
	MultiKPC-F	CATTCAAGGGCTTTCTTGCTGC	538
	MultiKPC-R	ACGACGGCATAGTCATTTGC	

^b^Y = T or C; R = A or G; S = G or C; D = A or G or T.

**Table 4:** Components of multiplex PCR master mix for the detection of carbapenemase genes (final volume 50 μl) and amplification conditions [31].

**Multiplex PCR pool**	**Primer name**	**Required primer’s concentration (pmol/50μl)**	**Added quantities of PCR master mix components (μl)**			**Amplification conditions**
			**Primer**	**DNA**	**Sterile D.W.**	1. Initial denaturation at 94°C for 10 min. 2. 30 cycles of: • denaturation at 94°C for 40s. • annealing at 55°C for amplification of *bla*_VIM_, *bla*_IMP_, *bla*_KPC_ genes, and 57°C for amplification of *bla*_GES_, and *bla*_OXA-48_ genes. • extension at 72°C for 1min; 3. Final elongation step at 72°C for 7 min.
PCR pool-I: *bla*_VEB_, *bla*_PER_ and *bla*_GES_	MultiGES-F & R	15 for each F and R primer	1 for each F and R primer	5	39	
	MultiPER-F & R					
	MultiVEB-F & R					
PCR pool-II: *bla*_GES_ and *bla*_OXA-48-like_	MultiGES-F & R	20 for each F and R primer			41	
	MultiOXA-48-F & R					
PCR pool-III: *bla*_IMP_, *bla*_VIM_, and *bla*_KPC_	MultiIMP-F & R	25 for each F and R primer			39	
	MultiVIM-F & R					
	MultiKPC-F	25				
	MultiKPC-R	10				

### Statistical analysis

Differences in the distributions of the studied determinants were tested by Chi square (SPSS software, version 2.1, IBM, NC, USA). A P value of ≤ 0.05 was considered to indicate statistical significance.

## Results and discussion

### Prevalence of *E*. *coli* among uropathogens

Of 1003 urine samples (one sample per patient) only 359 (35.7%) were positive for bacterial culture, where 168 (46.7%) of them were positive for Gram-negative bacteria. All of these Gram-negative isolates were identified biochemically by API20E rapid test according to index provided by the manufacturer (BioMerieux, France).

Among all positive bacterial cultures, *E*. *coli* was one of the commonly isolated bacteria (112/359: 31.9%). *Escherichia coli* also was the most commonly isolated species among Gram-negative bacteria (112/168: 66.6%), followed by *K*. *pneumoniae* (28: 16.6%), *Proteus mirabilis* (*P*. *mirabilis*) and *Pseudomonas* spp. (11: 6.5%, for each) and *Enterobacter* spp. (6: 3.5%). These results were similar to those obtained by other Iraqi researchers such as that which was carried out in Kirkuk city by Alsamarai and Ali [32] where they showed 41.6% of urine samples were culture positive and *E*. *coli* was the predominantly isolated bacteria (57.7%), followed by *K*. *pneumoniae* (14.5%), and *Proteus* spp. (10.3%). In Baghdad, Ghaima *et al*. [33] reported that 57.9% specimens were positive for bacterial culture and *E*. *coli* was the most common bacteria (34.0%) followed by *Klebsiella* spp. (14.6%); *Proteus* spp. (4.5%); *Pseudomonas* spp. (3.7%)and *Enterobacter* sp. (1.4%). Also, similar results were reported from other countries as those achieved by Rafalskiy *et al*. [34] in the Russian Federation where they found that 64.2% of the isolated uropathogens were Gram-negative and *E*. *coli* was the most prevalent (49.1%), followed by *K*. *pneumoniae* (9.5%), *P*. *mirabilis* (2.9%), *P*. *aeruginosa* (1.7%) and *Enterobacter* spp. (1.0%). In Grenada, Sharma *et al*. [35] showed that 65.4% of the isolates were Gram-negative bacteria with *E*. *coli* being the frequently isolated species (51.5%), followed by *K*. *pneumoniae* (20.0%) and *P*. *mirabilis* (10.0%). The high predominance of *E*. *coli* in patients with UTI is expected and it is well known by physicians and researchers all over the world as this bacterium represents a normal component of the intestinal microbiota of humans and animals and has strains with the potential of causing UTI and other extraintestinal infections. These UPEC strains have virulence traits that allow their successful colonization. So that, these virulence traits are considered the most important features differentiating the ExPEC from commensal and enteric *E*. *coli* [36]. Uropathogenic *E*. *coli* exhibit various virulence-associated factors (VFs) that assist them in attaching to, invading, and injuring the host. Among these virulence factors are adhesins (e.g. fmbriae), siderophores, iron-acquisition systems, capsules, toxins (e.g. haemolysin), invasins, and serum resistance associated proteins [36, 37].

### Phylogroups of *E*. *coli* isolates

In the present study, a quadraplex PCR assay developed by Clermont *et al*. [17] was applied to detect phylogroups’ reference of *E*. *coli* isolates (n = 112). The highest frequency of this study’s isolates was in group B2 (33.9%), followed by group A (24.1%), group D (17.8%), group B1 (8.0%), group C (4.4%) and group F (3.5%), whereas group E was not detected in any isolate. In addition, 9 isolates (8.0%) were non-typeable (Table 5). This predominance of phylogroup B2 among UPEC isolates is consistent with other studies conducted by Iraqi researchers including Merza and Jubrael [38] in Duhok city and Ahmed *et al*. [39] in Baghdad, who demonstrated that group B2 was the most frequently recovered phylogroup (56.7% and 36.0%, respectively). Furthermore, in ThiQar city, Hussain and Saleh [40] showed that 74% of UPEC belonged to phylogenetic group B2. Also, studies from different countries indicated that this group was the most prevalent among UPEC isolates. Among these studies, Iranpour *et al*. [41] in Iran, Massot *et al*. [42] in the Paris area, Miranda-Estrada *et al*. [43] in Mexico, Katongole *et al*. [44] in Uganda and Zhong *et al*. [45] in China, reported that group B2 had the highest frequency (39.3%, 34.0%, 42%, 40% and 16.7%, respectively) among UPEC isolates. This predominance of group B2 among UPEC isolates may be attributed to the fact that most virulence factors and antibiotic resistance genes existed jointly within this group and this could enhance survival fitness in urinary tract as recognized by many researchers such as Johnson *et al*. [46]; Lee *et al*. [47]; Najafi *et al*. [48]; Lara *et al*. [49] and Ali *et al*. [50].

**Table 5:** Distribution of *E*. *coli* phylogroups among 112 UPEC isolates from outpatients with acute UTI.

Phylogroup	*E*. *coli* isolates (n = 112)	
	No.	%
B2	38	33.9
B1	9	8.0
A	27	24.1
D	20	17.8
F	4	3.5
C	5	4.4
E	0	0
Nontypable (NT)	9	8.0

### Resistance of the B2 *E*. *coli* isolates to antimicrobials

Susceptibility to 17 antimicrobials was performed for all of the B2 UPEC isolates (*n* = 38). Resistance to *β*-lactams was the most common except carbapenems (imipenem and meropenem) to which all of the isolates were sensitive. High resistance rates were also found against tetracycline, trimethoprim-sulfamethoxazole, and nalidixic acid. Whereas, the isolates showed high sensitivity to ciprofloxacin, gentamicin, nitrofurantoin and amikacin. (Table 6). Recently, similar resistance patterns were published by Iraqi researchers [23].

**Table 6:** Antimicrobial resistance of group B2 *E*. *coli* isolates from outpatients with UTI.

Antimicrobial category	Antimicrobial agent	No. (%) of isolates* (n = 38)
Penicillins	Ampicillin (AMP)	38 (100)
Penicillins + β-lactamase inhibitors	Amoxicillin-clavulanic acid (AMC)	34 (89.4)
3rd and 4th generation cephalosporins	Cefepime (FEP)	32 (84.2)
	Ceftazidime (CAZ)	30 (78.9)
	Cefotaxime (CTX)	28 (73.6)
	Ceftriaxone (CRO)	25 (65.7)
Monobactams	Aztreonam (ATM)	25 (65.7)
Cephamycins	Cefoxitin (FOX)	15 (39.4)
Carbapenems	Imipenem (IPM)	0
	Meropenem (MEM)	0
Tetracyclines	Tetracycline (TE)	28 (73.6)
Trimethoprim	trimethoprim-sulfamethoxazole (SXT)	21 (55.2)
Quinolones	Nalidixic acid (NA)	17 (44.7)
	Ciprofloxacin (CIP)	10 (26.3)
Aminoglycosides	Gentamicin (CN)	7 (18.4)
	Amikacin (AK)	1 (2.6)
Nitrofurans	Nitrofurantoin (F)	3 (7.8)

*Resistant and intermediate.

Antimicrobial resistance in UPEC is a major concern in both humans and animals at a worldwide scale due to its increased resistance to several antibiotics [51], and expanding resistance to different classes of antimicrobial agents generally [52]. Indiscriminate and widespread use of antibiotics in addition to the practice of prescribing antibiotics to treat UTI without bacterial characterization led to increased resistance among uropathogens and to decreased effectiveness of oral therapies [5, 53], which gave an alarming level of antimicrobial resistance developing in UTI pathogens. Thus, rapid initiation of appropriate empirical treatment requires a good knowledge of epidemiological data concerning the sensitivity of uropathogens to antibacterial agents [54]. Furthermore, plasmids harboring resistance determinants can be transferred between bacteria, even between species, leading to the acquisition of resistance to new antibiotics via the emergence of mutant strains [55]. Also, some bacteria produce multiple *β*-lactamases, which may reduce the efficiency of *β*-lactam/*β*-lactamases inhibitor combination [5]. The rapid development of resistance to *β*-lactam antibiotics attributed to the emergence of ESBLs in the enteric bacteria [56]. This may be due to the excessive use of expanded spectrum cephalosporins (ESC) during clinical practice, where several studies have found a relationship between third-generation cephalosporins use and acquisition of ESBL-producing strains [57]. Therefore, the limited use of these antibiotics might be helpful to inhibit/avoid the emerging or spreading of multidrug-resistant Gram-negative bacteria [58]. One hundred percent sensitivity to imipenem and meropenem was observed among all B2 UPEC isolates investigated in this work. For bacterial infections, these antibacterials are the most reliable last-resort treatment [59]. Furthermore, the low resistance rates against aminoglycosides (gentamicin: 18.4% and amikacin: 2.6%) reported in the present study may be attributed to the rare use of these antibiotics in AL-Kut hospitals which may be due to their high costs in comparison with *β*-lactams [57]. Nitrofurantoin resistance was also noted in 7.8% of *E*. *coli* clinical isolates, and this may be due to the lower frequency use of this drug.

In addition, 100% of this study included isolates were MDR, while none of them were XDR or PDR. This may be due to the fact that *E*. *coli* pathogens have developed resistance to every class of antibiotics introduced to treat human and animal infections [60], and these infections are particularly challenging to treat [50]. This high prevalence of MDR among the isolates of the present study is alarming and necessitate the need for the clinicians to ensure the use of appropriate antibiotics for recommended periods in adequate doses in order to prevent emergence of multidrug resistant organisms. Many factors may have contributed to such high rates of resistance including misuse of antibiotics by health care professionals or non-skilled practitioners, misuse of antibiotics by the general public and inadequate surveillance due to lack of information arising from routine antimicrobial susceptibility testing, like reports from other developing countries. Careless usage of antibiotics is the most important factor that facilitates the development of MDR, which triggers the selection and distribution of antibiotic-resistant pathogens in clinical practice [56, 61]. Iraq is one of the developing countries where antibiotics are sold over the counter, an attitude that encourages self-medication. On the other hand, it is remarked that during period, a group of antibiotics become more used than others without susceptibility tests, which may lead to variability in their resistance [62]. Other factors known to influence the evolution and transfer of MDR among microorganisms are incomplete doses, ease of access, over prescribing antibiotics without laboratory results and indiscriminate use of antimicrobials in agriculture and livestock sectors [61]. Antibiotics resistance arises quickly and spreads rapidly, especially when resistance genes are horizontally transferred via plasmids and integrons among individuals, among species, and even among bacterial kingdom [63]. Much of the problem of antimicrobial resistance has been shown to be due to the presence of transferable plasmids encoding MDR and their dissemination among different enterobacterial species and it is common for a single plasmid to simultaneously mediate resistance to multiple antimicrobials and to be shared among different bacterial genera [64].

These MDR isolates showed resistance to most antimicrobials tested in this study except carbapenems (all isolates were sensitive), amikacin (2.6%) and nitrofurantoin (7.8%). These results ensure that continuous and inappropriate use of antimicrobials is a major risk factor for development of resistance. It was realized that the inappropriate use of antimicrobials has been shown to play a pivotal role in the emergence of MDR organisms [65, 66]. Also, MDR is largely associated with ESBLs’ production as mentioned by several researchers [64, 65, 67–69].

### Distribution of carbapenemase genes among the isolates

**Fig 1:**
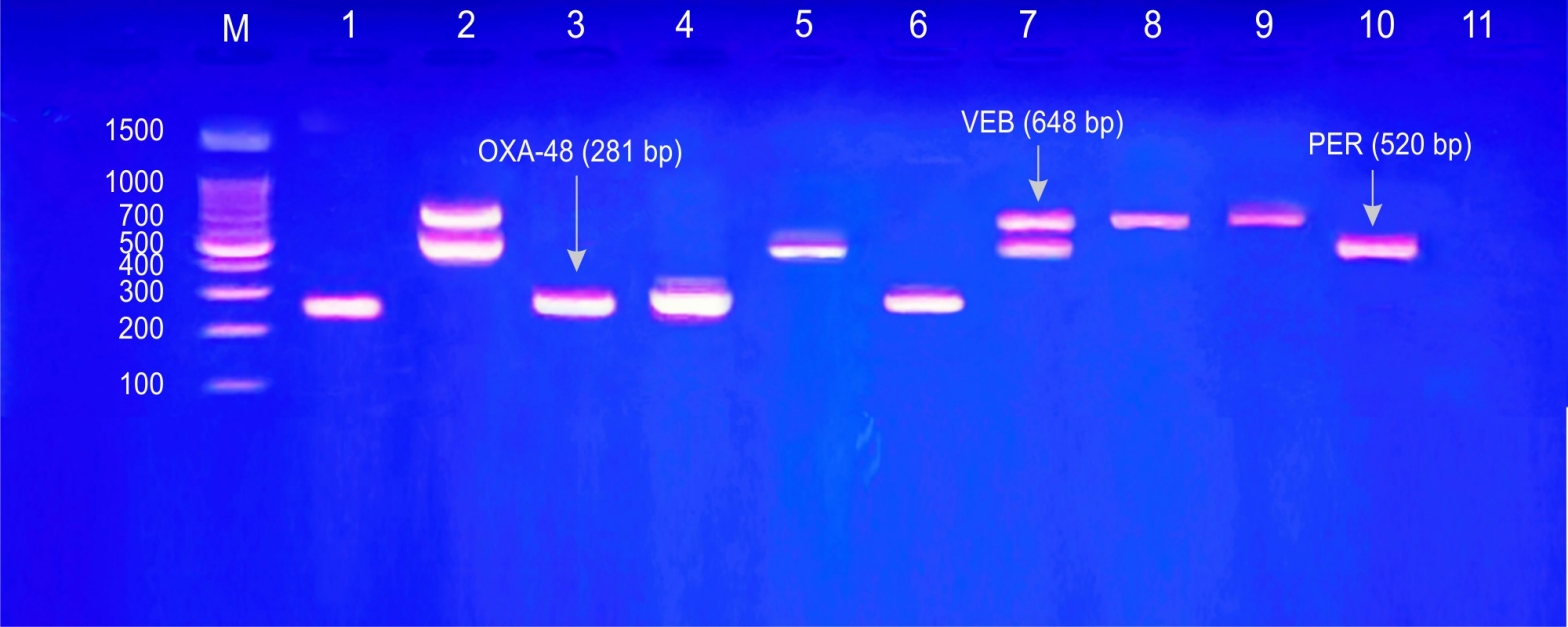
Gel electrophoresis of PCR amplified products for detection of carbapenemases genes: Multiplex I & II. Multiplex I & II: *bla*_GES_ (399 bp), *bla*_PER_ (520 bp), *bla*_VEB_ (648 bp) and *bla*_OXA-48_ (281 bp). Lane m: DNA Ladder (100 pb); Lanes 1, 3, 4 and 6: positive results for *bla*_OXA-48_; Lanes 2 and 7: positive results for *bla*_VEB_ and *bla*_PER_; Lanes 5 and 10: positive results for *bla*_PER_; Lanes 8 and 9: positive results for *bla*_VEB_; Lane 11: negative results for all genes.

**Fig 2:**
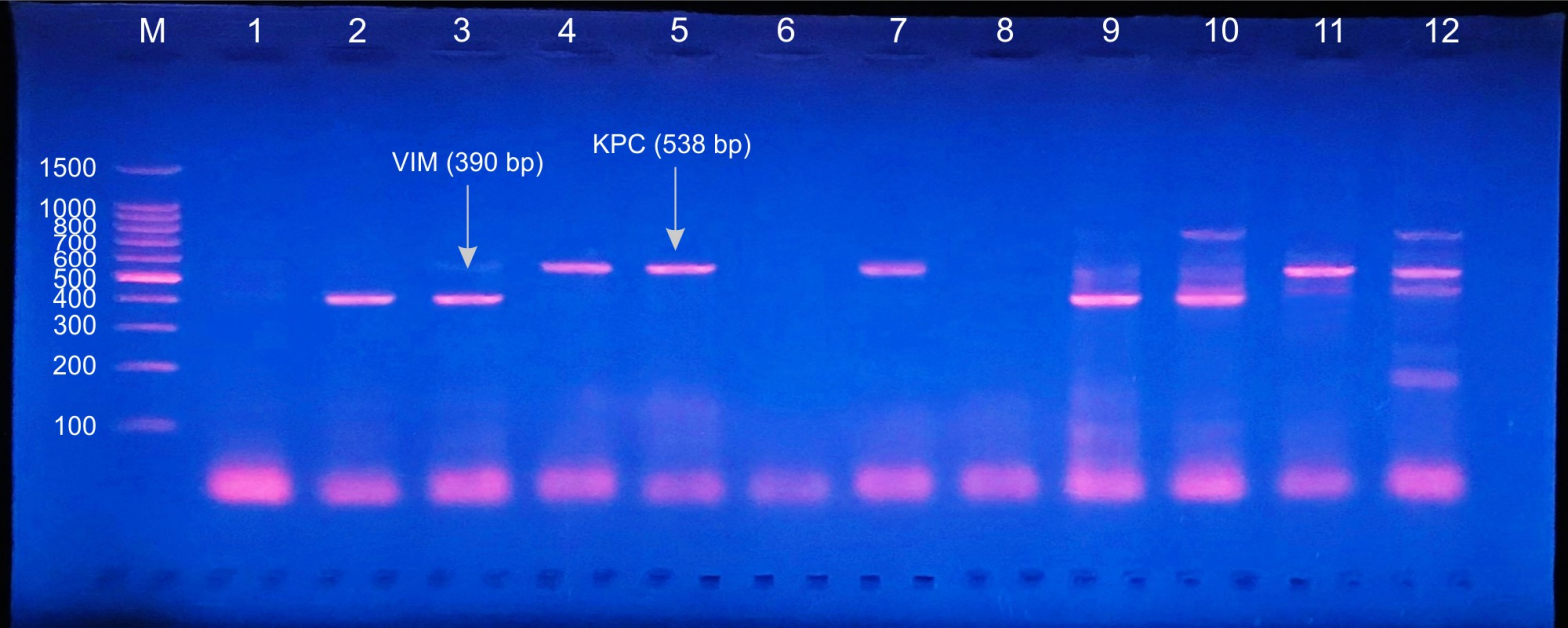
Gel electrophoresis of PCR amplified products for detection of carbapenemases genes: Multiplex III. Multiplex III: *bla*_IMP_ (139 bp), *bla*_VIM_ (390 bp) and *bla*_KPC_ (538 bp). Lane m: DNA Ladder (100 pb); Lanes 1, 6 and 8: negative results for *bla*_IMP_, *bla*_VIM_ and *bla*_KPC_; Lanes 2, 3, 9 and 10: positive results for *bla*_VIM_; Lanes 4, 5, 7, 11, and 12: positive results for *bla*_KPC_.

All of this study included B2 *E*. *coli* isolates were ESBLs producers with predominance of CTX-M-1 (unpublished data). In this work, investigated carbapenemase genes included: *bla*_OXA-48_, *bla*_PER_, *bla*_KPC_, *bla*_VEB_, *bla*_VIM_, *bla*_GES_ and *bla*_IMP_ (Figs 1 and 2). Out of 38 B2 UPEC isolates, 29 (76.3%) were carbapenemase producers. Of these carbapenemases, *bla*_OXA-48_ gene was the most frequent (57.8%), followed by *bla*_PER_ (47.3%), *bla*_KPC_ (15.7%), *bla*_VEB_ and *bla*_VIM_ (10.5%, for each). Whereas, no PCR-amplification products were noticed with *bla*_GES_ and *bla*_IMP_ genes (Table 7).

**Table 7:** Distribution of carbapenemase genes among B2 *E*. *coli* isolates from outpatients with acute UTI.

Carbapenemase gene	No. (%) of isolates (n = 38)
*bla*_OXA-48_	22 (57.8)
*bla*_PER_	18 (47.3)
*bla*_KPC_	6 (15.7)
*bla*_VEB_	4 (10.5)
*bla*_VIM_	4 (10.5)
*bla*_GES_	0
*bla*_IMP_	0

Also, coproduction of these enzymes occurred among 17 isolates. The combination of *bla*_OXA-48_ and *bla*_PER_ was the most frequent (41.1%) (Table 8). Antimicrobial susceptibility of these carbapenemase producers was depicted in Table 9.

**Table 8:** Coproduction of carbapenemase genes by B2 *E*. *coli* isolates from outpatients with acute UTI.

Combination of Carbapenemase genes	No. (%) of isolates (n = 17)
***bla***_***OXA-48***_ **+ *bla***_**PER**_	**7 (41.1)**
***bla***_**OXA-48**_ **+*bla***_**VIM**_	**1 (5.8)**
***bla***_**OXA-48**_ **+*bla***_**KPC**_	**1 (5.8)**
***bla***_**VEB**_ **+*bla***_**PER**_	**1 (5.8)**
***bla***_**OXA-48**_ **+ *bla***_**PER**_ **+ *bla***_**KPC**_	**3 (17.6)**
***bla***_**OXA-48**_ **+ *bla***_**VIM**_ **+ *bla***_**KPC**_	**1 (5.8)**
***bla***_**OXA-48**_ **+ *bla***_**PER**_ **+ *bla***_**VEB**_	**1 (5.8)**
***bla***_**OXA-48**_ + ***bla***_**PER**_ **+ *bla***_**KPC**_ **+ *bla***_**VEB**_	**1 (5.8)**
***bla***_**OXA-48**_ **+ *bla***_**PER**_ **+*bla***_**VIM**_ **+*bla***_**VEB**_	**1 (5.8)**

**Table 9:** Antimicrobial resistance of carbapenemase producing B2 UPEC isolates.

Antimicrobial category	Antimicrobial agent	No. (%) of isolates* (n = 29)
Penicillins	Ampicillin (AMP)	29 (100)
Penicillins + β-lactamase inhibitors	Amoxicillin-clavulanic acid (AMC)	27 (93.1)
3rd and 4th generation cephalosporins	Cefepime (FEP)	24 (82.7)
	Ceftazidime (CAZ)	24 (82.7)
	Cefotaxime (CTX)	23 (79.3)
	Ceftriaxone (CRO)	21 (72.4)
Monobactams	Aztreonam (ATM)	21 (72.4)
Cephamycins	Cefoxitin (FOX)	10 (34.4)
Carbapenems	Imipenem (IPM)	0
	Meropenem (MEM)	0
Tetracyclines	Tetracycline (TE)	21 (72.4)
Trimethoprim	trimethoprim-sulfamethoxazole (SXT)	19 (65.5)
Quinolones	Nalidixic acid (NA)	16 (55.1)
	Ciprofloxacin (CIP)	9 (31)
Aminoglycosides	Gentamicin (CN)	7 (24.1)
	Amikacin (AK)	1 (3.4)
Nitrofurans	Nitrofurantoin (F)	3 (10.3)

*Resistant and intermediate.

From these results, it seems likely that the situation is more complicated than it is expected as the isolates: (1) belonged to the highly virulent phylogroup B2 and (2) carried not only one gene but combination of genes in addition to ESBLs’ production. This study results revealed that all carbapenemase producers (100%) were MDR. Patterns of antimicrobial resistance and carbapenemase genes of these isolates were presented in Table 10. Multidrug resistance is not attributed to a certain set of genes but in fact it is a result of possession of a combination of different genes especially ESBLs. Nikaido [70] concluded that MDR is due to accumulation of genes, each coding for resistance to a single antimicrobial agent, on R plasmids. This accumulation of these genes is carried out by transposons, integrons, and insertion sequences (ISCR). In UPEC, the mechanisms of antimicrobial resistance are diverse and comprise: production of inactivating enzymes either hydrolytic enzymes such as β-lactamases or no hydrolytic enzymes such as aminoglycoside acetyl transferase enzymes. Other mechanisms include: active efflux pumps, alteration of target site, horizontal gene transfer by insertion sequences, gene cassettes, integrons, and transposons [3, 71]. Eshetie *et al*. [72] reported that carbapenemase producing Enterobacteriaceae had significant drug resistance rates compared to other MDR Enterobacteriaceae and that production of these enzymes is one of the main mechanisms in the occurrence of drug resistance in this family. So that, Jain *et al*. [73] concluded that antimicrobial resistance due to β-lactamases is emerging as a major problem in UTI; routine surveillance of these β -lactamases will help in control of treatment failures. In fact, one of the critical priorities of the World Health Organization is carbapenem-resistant ESBL-producing bacterial pathogens for which new antibiotics should be designed [4].

**Table 10:** Phenotypic and genotypic resistance patterns of 29 MDR carbapenemase producing B2 UPEC isolates.

No. (%) of isolates	Phenotypic resistance*	carbapenemase genes
6 (20.6)	CTX, CRO, CAZ, FEP, ATM, AMP, AMC, SXT, TE, CIP, NA	*bla*_OXA-48_, *bla*_VIM_, *bla*_KPC_, *bla*_PER_, *bla*_VEB_
3(10.3)	CTX, CRO, CAZ, FEP, ATM, AMP, AMC, TE	*bla*_PER_, *bla*_OXA-48_, *bla*_KPC_
2 (6.8)	CTX, CRO, CAZ, FEP, ATM, AMP, AMC, CN, SXT, TE, F, CIP, NA	*bla*_PER_, *bla*_OXA-48_, *bla*_KPC_
2 (6.8)	CAZ, FEP, FOX, AMP, AMC, TE	*bla*_VIM_, *bla*_PER_, *bla*_OXA-48_, *bla*_KPC_
1 (3.4)	CTX, CRO, CAZ, FEP, FOX, ATM, AMP, AMC, CN, SXT, NA	*bla*_PER_, *bla*_OXA-48_
1(3.4)	CTX, CRO, CAZ, FEP, ATM, AMP, AMC, CN, SXT, TE, CIP, NA	*bla*_PER_, *bla*_OXA-48_
1(3.4)	CTX, CRO, CAZ, FEP, FOX, ATM, AMP, AMC, SXT, TE, CIP, NA	*bla*_PER_, *bla*_VEB_, *bla*_OXA-48_
1 (3.4)	CTX, CRO, CAZ, FEP, FOX, ATM, AMP, AMC, CN, CIP, NA	*bla*_PER_
1 (3.4)	CTX, CAZ, FEP, FOX, AMP, AMC, AK, SXT, TE	*bla*_PER_
1 (3.4)	CTX, CRO, CAZ, FEP, FOX, ATM, AMP, AMC, SXT, TE	*bla*_PER_, *bla*_OXA-48_
1 (3.4)	CTX, CRO, CAZ, FEP, FOX, AMP, AMC, SXT, F, CIP, NA	*bla*_OXA-48_
1 (3.4)	CTX, CRO, CAZ, FEP, ATM, AMP, AMC, CN, SXT, TE	*bla*_PER_, *bla*_OXA-48_, *bla*_KPC_
1(3.4)	CTX, CRO, CAZ, FEP, FOX, ATM, AMP, AMC, TE	*bla*_PER_, *bla*_VEB_
Continued		
1 (3.4)	CTX, CRO, CAZ, FEP, ATM, AMP, AMC, SXT, TE	*bla*_PER_, *bla*_VEB_, *bla*_OXA-48_, *bla*_VIM_
1 (3.4)	AMP, AMC, SXT, TE, NA	*bla*_OXA-48_
1 (3.4)	AMP, AMC, CN, SXT, NA	*bla*_PER_, *bla*_OXA-48_
1 (3.4)	CAZ, AMP, AMC, SXT, TE	*bla*_PER_
1 (3.4)	(CTX, CRO, CAZ, FEP, ATM, AMP, AMC	*bla*_PER_
1 (3.4)	CTX, ATM, AMP, NA	*bla*_OXA-48_
1 (3.4)	FEP, FOX, AMP	*bla*_PER_

* Cephalosporins (CTX: cefotaxime, CRO: ceftriaxone, CAZ: ceftazidime, FEP: cefepime); Monobactams (ATM: aztreonam); Penicillins (AMP: ampicillin); Penicillins + *β*-lactamase inhibitors (AMC: amoxicillin-clavulanic acid); Trimethoprim (SXT: trimethoprim-sulfamethoxazole); Tetracyclines (TE: tetracycline), Quinolones (CIP: ciprofloxacin, NA: nalidixic acid). Aminoglycosides (CN: gentamicin, AK; amikacin; Nitrofurans (F: nitrofurantoin), Cephamycins (FOX: cefoxitin).

In a recent study [23] that was carried out in Iraq, it was found that out of 300 isolates, 11 (3.66%) of them were phenotypically resistant to carbapenems, whereas only 3 (1%) isolates were genotypically positive for carbapenemases of which *bla*_OXA-48_ and *bla*_IMP_ genes were co-existed in these 3 isolates, while *bla*_KPC_, *bla*_NDM_ and *bla*_VIM_ were not found. Furthermore, nine of these 11 isolates belonged to B2 phylogroup and two were from B1 group. Most other studies [20–22, 74] showed the predominance of OXA-48 among *E*. *coli* isolates, especially B2 isolates. Other studies in Iraq [75] and around the world [44, 76–82] also revealed the occurrence of different carbapenemase genes among *E*. *coli* isolates.

Although, all of the isolates in this study were phenotypically susceptible to imipenem and meropenem by disc diffusion method, genotypically, carbapenemase production was obvious among them ranging from 10.5% for both VEB and VIM to 57.8% for OXA-48. This discrepancy was noticed by other researchers [20, 74]. It was explained that detection of carbapenemase-producing *E*. *coli* (CP- *E*. *coli*) isolates is often difficult due to low carbapenem MICs that may remain within the susceptibility range [83]. Also, OXA-48 enzymes are known to exhibit only low hydrolytic activity toward carbapenems [84]. Moreover, it seems better to use ertapenem disc diffusion for phenotypic detection of those especially producing OXA-48 rather than imipenem or meropenem [20]. The same researchers concluded that laboratory detection of CP-E. *coli* may be more difficult in comparison with CP-*K*. *pneumoniae*, particularly in the case of OXA-48, because: (i) the isolates may appear susceptible to imipenem and meropenem; and (ii) there is a high frequency of OXA-48-producing isolates without ESBL co-production.

The results of the present investigation showed high occurrence of carbapenemase genes among B2 UPEC isolates. This may be due to increased use of carbapenems by physicians for treatment of serious and even non-serious cases in the study area (personal communication). Also, in this work only B2 isolates were investigated for carbapememases among which these genes are concentrated. In spain [20], it was revealed that the CP-*E*. *coli* occurred after a continuous increase in resistance to third-generation cephalosporins and fluoroquinolones.

The production of all kinds of carbapenemases by *E*. *coli* isolates represent a major issue with further problem in UTI treatment [60]. From a therapeutic perspective, CRE represent a threat as only a few antibiotics retain activity against them. This is due to the ability of carbapenemases to hydrolyze most other β-lactam antibiotics, and to frequent coexistence in CRE isolates of additional mechanisms of resistance against other antibiotics such as fluoroquinolones and aminoglycosides [85]. Furthermore, the indiscriminate use of carbapenems can select resistance to these main drugs and sow seeds for significant therapeutic problems that may occur in the future. Efficient infection-control methods for outbreak management are also needed; and prevention approaches, e.g., antibiotic rotation, are needed to reduce the selection and spread of these highly resistant pathogens [86]. Today, the extensive international movement and exchange has helped OXA-48 producing Enterobacteriaceae to spread from many Middle-Eastern countries into other parts of the world [11].

## Conclusions

This study revealed high occurrence of carbapenemase genes and MDR among phylogroup B2 UPEC isolates from outpatients with acute UTI in Wasit Province, Iraq. These findings may indicate the concentration of these genes among *E*. *coli* phylogroup B2 members. Also, this study included isolates showed high resistance rates to most antimicrobials used in this work except aminoglycosides and nitrofurantoin, so that, it is strongly recommended to use aminoglycosides and nitrofurantoin for empirical treatment of UTIs in the study area.

## Supporting information

S1 Raw images (PDF)
