# Supplementary figures and images for: Prevalence of common carbapenemase genes and multidrug resistance among uropathogenic Escherichia coli phylogroup B2 isolates from outpatients in Wasit Province/ Iraq
Source: PLoS One. 2022 Jan 25;17(1):e0262984. doi: 10.1371/journal.pone.0262984 (PMC8789106; doi:10.1371/journal.pone.0262984)

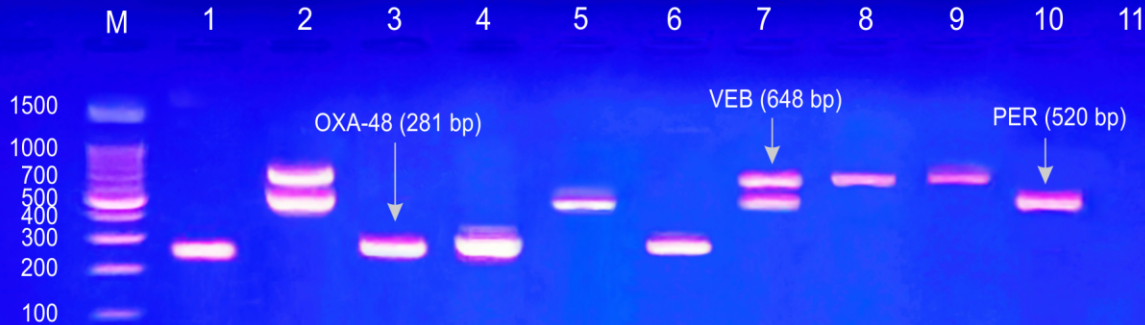

**Fig 1**

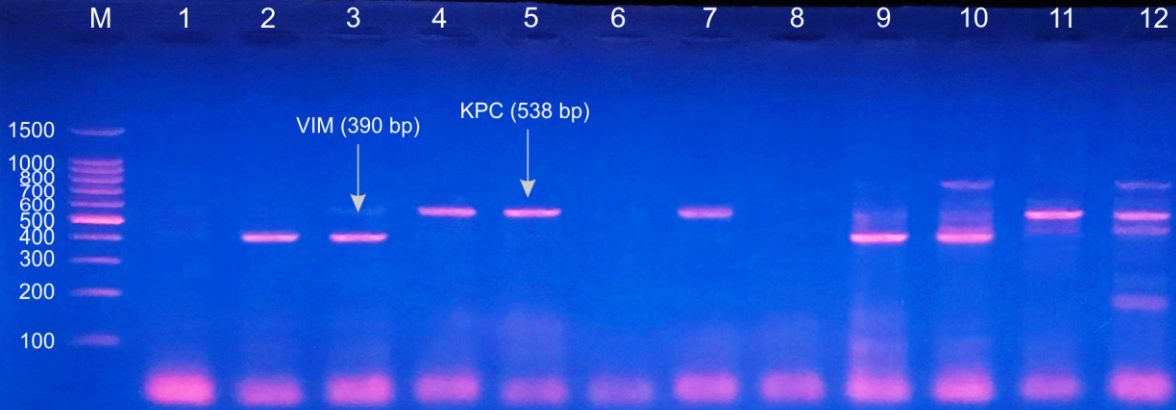

**Fig 2**

Supplement: S1 Raw images — (PDF) [file pone.0262984.s001.pdf]
